# The effect of general anesthesia on the test–retest reliability of resting-state fMRI metrics and optimization of scan length

**DOI:** 10.3389/fnins.2022.937172

**Published:** 2022-08-16

**Authors:** Faezeh Vedaei, Mahdi Alizadeh, Victor Romo, Feroze B. Mohamed, Chengyuan Wu

**Affiliations:** ^1^Department of Radiology, Jefferson Integrated Magnetic Resonance Imaging Center, Thomas Jefferson University, Philadelphia, PA, United States; ^2^Department of Neurological Surgery, Vickie and Jack Farber Institute for Neuroscience, Thomas Jefferson University, Philadelphia, PA, United States; ^3^Department of Anesthesiology, Thomas Jefferson University, Philadelphia, PA, United States

**Keywords:** resting-state, fMRI, anesthesia, test–retest reliability, scan length

## Abstract

Resting-state functional magnetic resonance imaging (rs-fMRI) has been known as a powerful tool in neuroscience. However, exploring the test–retest reliability of the metrics derived from the rs-fMRI BOLD signal is essential, particularly in the studies of patients with neurological disorders. Here, two factors, namely, the effect of anesthesia and scan length, have been estimated on the reliability of rs-fMRI measurements. A total of nine patients with drug-resistant epilepsy (DRE) requiring interstitial thermal therapy (LITT) were scanned in two states. The first scan was performed in an awake state before surgery on the same patient. The second scan was performed 2 weeks later under general anesthesia necessary for LITT surgery. At each state, two rs-fMRI sessions were obtained that each one lasted 15 min, and the effect of scan length was evaluated. Voxel-wise rs-fMRI metrics, including the amplitude of low-frequency fluctuation (ALFF), the fractional amplitude of low-frequency fluctuation (fALFF), functional connectivity (FC), and regional homogeneity (ReHo), were measured. Intraclass correlation coefficient (ICC) was calculated to estimate the reliability of the measurements in two states of awake and under anesthesia. Overall, it appeared that the reliability of rs-fMRI metrics improved under anesthesia. From the 15-min data, we found mean ICC values in awake state including 0.81, 0.51, 0.65, and 0.84 for ALFF, fALFF, FC, and ReHo, respectively, as well as 0.80, 0.59, 0.83, and 0.88 for ALFF, fALFF, FC, and ReHo, respectively, under anesthesia. Additionally, our findings revealed that reliability increases as the function of scan length. We showed that the optimized scan length to achieve less variability of rs-fMRI measurements was 3.1–7.5 min shorter in an anesthetized, compared to a wakeful state.

## Introduction

Resting-state functional magnetic resonance imaging (rs-fMRI) estimates blood oxygen level-dependent (BOLD) signal fluctuations at low frequency (<0.1 Hz) correspond to synchronized variations in spontaneous neuronal activity in the resting brain. The ability to measure brain functional connectivity (FC) non-invasively and with no instructed task has invaluable utility in clinical application and neuroscience research in animal and human populations. However, FC is not static, and the connectivity strengths may alter during a single session or between sessions' resting-state scanning. Thus, despite its potential, rs-fMRI suffers from significant variabilities involved in the BOLD signal that may cause difficulties in the replication of results across studies (Mason et al., 2007; Patriat et al., 2013). Several factors can contribute to the intra-subject variabilities and affect the reliability of estimated brain FC including head motion, physiological noise such as cardiac and respiratory effects, MRI acquisition parameters, and data analysis/standardization strategies (Birn, 2012; Guo et al., 2012; Zuo and Xing, 2014; Finn et al., 2017; Parkes et al., 2018; Wang et al., 2021).

General anesthesia has been used in animal and human studies to reduce the potential of motion artifacts and fluctuations in behavior in order to generate a consistent mental state and increase the control of certain aspects of a subject's physiology (Wu et al., 2016; Levine et al., 2020). At the same time, it has been shown that general anesthesia including intravenous and volatile anesthetics suppresses neuronal activity and affects fMRI BOLD response in both task-based and resting-state fMRI studies in a dose-dependent manner (Hemmings et al., 2005; Grandjean et al., 2014; Jonckers et al., 2014; Aksenov et al., 2015; Huang et al., 2016; Moody et al., 2021). Studies performed primarily in animals and humans have examined the effect of intravenous and volatile anesthetic agents on BOLD signal variability and demonstrated that anesthetics reduce the overall variations of intrinsic BOLD fluctuations and dynamical complexity (Huang et al., 2014, 2016; Bettinardi et al., 2015; Baria et al., 2018). Hence, we speculate that the BOLD signal and resultant brain FC measurement under this condition will be more consistent and reliable within-subjects and between-subjects over repeating experiments.

Additionally, scan duration has been known as another influential factor that affects the stability of rs-fMRI BOLD response. Recently, several studies have answered this question regarding how much data are needed to be acquired to achieve a stable and reliable resting-state BOLD response. They estimated optimized resting-state scan duration particularly using graph theory measurements (Wang et al., 2011; Termenon et al., 2016) and network-based FC (Birn et al., 2013; Liao et al., 2013). Low-frequency oscillations (LFOs) of the brain are one of the approaches that have been used in neuroscience to measure neuronal activation at rest. LFOs of the rs-fMRI signals reflect spontaneous brain neural activity by employing simple spectral filtering within a range of ~0.01–0.1 Hz. It has been shown to have encouraging test–retest reliabilities especially for the measures derived from gray matter since this is less affected by physiological factors (Zuo et al., 2010; Tong et al., 2019; Vedaei et al., 2021). The indices including the absolute and fractional amplitude of low-frequency fluctuations (ALFF and fALFF) and regional homogeneity (ReHo) have been introduced to measure LFO amplitude. ALFF and fALFF are the fast Fourier transform (FFT)-based indices of LFO amplitude. They are defined as the absolute and relative magnitude of spontaneous fluctuations in BOLD signal and are assumed to reflect brain neural activity during rest (Zou et al., 2008; Yang et al., 2018). Likewise, ReHo detects the local synchronization of LFOs using Kendall's coefficient of concordance. It represents the similarity between the time series of a given voxel and that of its nearest neighbors (Zang et al., 2004; Jiang and Zuo, 2016; Specht, 2020; Vedaei et al., 2021). Statistical voxel-wise test–retest reliability has been used in recent rs-fMRI studies to estimate the reproducibility of measured brain FC due to its ability to explicitly model measurement's variance. However, fewer studies have employed it on LFO measurements including ALFF, fALFF, and ReHo in the same cohort (Zuo and Xing, 2014).

While prior studies investigated either the effect of general anesthesia or scan length in rs-fMRI in separate experiments, our study is the first human study combining these two influential factors to estimate the reliability of rs-fMRI measurements. The present study examined the impact of general anesthesia on the test–retest reliability of voxel-wise rs-fMRI metrics including ALFF, fALFF, ReHo, and FC. Second, we investigated the effect of resting-state scan length on the reliability of the measurements. We aimed to determine standardized scan lengths of rs-fMRI which ensure robust and reliable BOLD responses in both conditions of wakefulness and under anesthesia.

## Methods

### Participants

**Figure 1:**
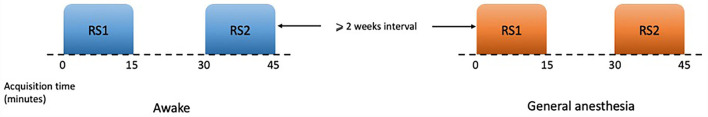
Schematic of resting-state paradigms. Each session of scan consisted of two sets of rs-fMRI which lasted 15 min, and at least 15 min gap between them. There was an interval of at least 2 weeks between two sessions of awake and under general anesthesia.

Candidates for this study included patients who presented to the Comprehensive Epilepsy Center at Thomas Jefferson University Hospitals with drug-resistant epilepsy (DRE) who have been deemed appropriate candidates for laser interstitial thermal therapy (LITT). All patients had a diagnosis of mesial temporal lobe epilepsy (mTLE) with unilateral mesial temporal sclerosis (MTS) according to the standard clinical criteria. All patients with mTLE with MTS were selected for the laser ablation of the amygdala–hippocampal complex. In order to deal with a typically complex patient cohort and often heterogeneous in seizure characteristics and clinical history, we had restricted the cohort through stringent inclusion/exclusion criteria: history of drug-resistant mTLE; on stable (anti-epileptic drugs) AEDs and compliant with medication use; an average of at least one complex partial or secondarily generalized seizure compatible with mTLE per month; seizure symptoms and/or auras compatible with mTLE; video EEG showed evidence of seizures from one temporal lobe consistent with mTLE; MRI had evidence consistent with mesial temporal lobe sclerosis. These patients were selected because as part of their routine clinical course they required an awake MRI for preoperative surgical planning, followed by an MRI under general anesthesia during the LITT procedure, with ~2 weeks in-between. All patients underwent the standard informed consent process before being included in the study. A total of nine patients (four men and five women, aged 28–60 years) were enrolled. Each patient completed two rs-fMRI sequences during each scanning session (~15 min apart) (Figure 1). During the resting-state MRI scanning, the subjects were instructed to lie down, close their eyes, and rest without thinking about a specific thing, but refrain from falling asleep. To minimize head movement, straps and foam pads were used to fix the head comfortably during the scanning. The study was approved by the institutional review board (IRB) of Thomas Jefferson University Hospital. All methods were performed in accordance with the relevant guidelines and regulations.

### Data acquisition

Both MRI sessions were performed on a 3.0T Achieva Phillips scanner with an eight-channel head coil. fMRI images were acquired axially using a single-shot echo planar imaging (EPI) sequence in the same anatomical location prescribed for T1-weighted images. The T1-weighted imaging parameters used were as follows: FOV = 24.0 × 24.0 cm^2^, voxel size = 1.0 × 1.0 × 1.0 mm^3^, matrix size = 352 × 352 × 150, TR = 7.5 ms, TE = 3.4 ms, and slice thickness = 1 mm. Functional MR imaging parameters were FOV = 23.0 × 23.0 cm^2^, voxel size = 3.5 × 3.5 × 3.5 mm^3^, matrix size = 128 × 128 × 34, TR = 2 s, TE = 25 ms, and number of averages = 1.

### Anesthesia administration

The second MRI scan was acquired during the LITT procedure and under general anesthesia. All patients were evaluated by an anesthesiologist and underwent institutional standard of pre-anesthetic preparation. Every patient received a standard induction of intravenous propofol (130–300 mg) 15–20 min before scanning. After endotracheal intubation, sevoflurane anesthesia was administered through the endotracheal tube and maintained with 0.6–1.2 mean alveolar concentration (MAC) and 100% fractional inspiratory oxygen (FIO2) during MRI acquisition. Mean arterial blood pressure was also maintained at 65–75 mmHg and end-tidal carbon dioxide (ETCO_2_) at 30–35 mmHg throughout the procedure. After MRI acquisition and LITT procedure were completed, patients were reversed with Sugammadex 2–4 mg/kg, extubated as per routine, and were observed in the post-anesthetic care unit during their recovery. Note that, the MRI scanning under anesthesia was acquired intraoperatively prior to the commencement of the ablation procedure.

### Data analysis

All rs-fMRI data were preprocessed using data processing and analysis for resting-state brain imaging (DPABI, V5.1_201201; http://rfmri.org/dpabi) (Yan et al., 2016) based on statistical parametric mapping (SPM12; http://www.fil.ion.ucl.ac.uk/spm) running on MATLAB R2020b (The Math Works, Inc., Natick, MA, USA). The pre-processing steps are listed as follows: the first 10 volumes were discarded to get the steady state MRI signal and adaptation of participants in the scanning environment. The remaining volumes were corrected for slice timing and head motion using six rigid body motion parameters. Head motion parameters were calculated. No one was excluded from the study because of the exclusion criteria of mean framewise displacement (FD) (Jenkinson) >0.5 mm (Supplementary material). Next, each individual T1-weighted structural image and the mean of the realigned EPI images were co-registered and normalized to the EPI template in Montreal Neurological Institute (MNI) space with a resampling voxel size of 3 × 3 × 3 mm. Due to the sensitivity of rs-fMRI measurements to micro head motions, the Friston 24-parameter model (the 24 parameters including six head motion parameters, six head motion parameters of the previous scan, and the 12 corresponding squared items) was applied to regress out the head motion effects from the realigned data. Additionally, regression analysis was conducted to minimize the effect of white matter and cerebrospinal fluid signal. Finally, the remaining images were filtered using a temporal band-pass of 0.01–0.08 Hz to reduce the effects of low-frequency drifts and high-frequency physiological noise.

All data processing steps were restricted within gray matter, for which a gray matter mask was created. Statistical parametric mapping 12 (https://www.fil.ion.ucl.ac.uk/spm/) was used to conduct the segmentation of gray matter. Then, the probabilistic maps were binarized using *fslmaths* tools (cutoff = 0.2) and were averaged to generate the gray matter mask.

### ALFF/fALFF calculation

The amplitude of low-frequency fluctuation (ALFF) measures the intensity of regional spontaneous brain activity at rest, and the fractional ALFF (fALFF) is felt to have higher sensitivity and specificity with less inclusion of artifacts from vascular signals. For each subject, before ALFF/fALFF calculation, spatial smoothing (Gaussian kernel of full-width half maximum, FWHM = 6 mm) was performed. For each voxel, the time series of the rs-fMRI signal was converted to the frequency range using a fast Fourier transform (FFT), and the square root of the power spectrum was measured at each frequency of the power spectrum and the averaged square root was obtained across the 0.01–0.08 Hz domain. This averaged square root is called ALFF. Then, fALFF was measured as the ratio of power in the low-frequency band (0.01–0.08 Hz) (ALFF) to the power of the entire frequency range (0–0.25 Hz). For the standardization purpose, for each subject, the ALFF/fALFF of each voxel was divided by the global mean of ALFF/fALFF within the gray matter mask to generate the mALFF/mfALFF maps (Zou et al., 2008; Zhou et al., 2014; Jia et al., 2020).

### ReHo calculation

ReHo measurement was performed after band-pass filtering (0.01–0.08 Hz). This is accomplished on a voxel-based basis by calculating Kendall's coefficient of concordance (KCC) for a given time series that is assigned as the center voxel with those of its nearest 26 neighboring voxels (Equation 1) (Zang et al., 2004).


(1) $$\begin{array}{c} w\;=\;\frac{\sum^{\text{​}}(R_{i})-n{\left({\bar{R}}_{i}\right)}^{2}}{\frac{1}{12}K^{2}(n^{3}\;-n)\;} \end{array}$$

In this formula, *w* is the KCC (range from 0 to1) among given voxels; *K* is the number of neighboring voxels (*K* = 26); *R*_*i*_ is the mean rank across nearest neighbors (26 voxels) at the *ith* time point; and *n* is the total number of time points. To avoid any artificial coherence due to spatial smoothing, ReHo was calculated before spatial smoothing. For the standardization purpose, the ReHo value at each voxel was divided by the global mean of ReHo within the gray matter mask to obtain the mReHo maps. Spatial smoothing with an isotropic Gaussian kernel of 6 mm full-width half-maximum (FWHM) was performed after the ReHo calculation.

### Voxel-wise functional connectivity (FC)

For each subject, voxel-wise FC was measured by estimating Pearson's correlations between the time series of any pairs of brain voxels which resulted in individual zFC matrix within the gray matter mask. Then, for given voxel i, FC was measured using the equation as follows (Equation 2):


(2) $$\begin{array}{l} \text{FC (i) =}\frac{1}{N_{voxels}\;-1}\underset{j\neq i}{\sum }Z_{ij},\;r_{ij}>r_{0} \end{array}$$

where *z*_*ij*_ is the Fisher's Z-transformed version of the correlation coefficient, *r*_*ij*_, between voxel *i* and voxel *j*, and *r*_0_ is a correlation threshold that is used to exclude weak correlations possibly arising from noises (*r*_0_= 0.2 in this study). *r*_*ij*_ is converted to *z*_*ij*_ using Fisher's Z-transformation. *N*_*voxels*_ is also defined as the total number of voxels within the gray matter mask (*N*_*voxels*_ = 544,833) (Wang et al., 2014; Dai et al., 2015).

### Reliability of rs-fMRI metrics

Test–retest reliability has been shown to reflect the stability of a test measure under repeated experiments. Intraclass correlation coefficient (ICC) has been commonly used in estimating the test–retest reliability (Shrout and Fleiss, 1979; Somandepalli et al., 2015; Noble et al., 2019). In this study, resting-state fMRI reliability of the metrics including ALFF, fALFF, ReHo, and FC was estimated using ICC for awake and under anesthesia states according to the following equation (Equation 3) (Shrout and Fleiss, 1979):


(3) $$\begin{array}{l} \text{ICC =}\frac{MS_{b}\;-MS_{w}}{MS_{b}+\left(K-1\right)MS_{w}} \end{array}$$

where *MS*_*b*_ represents the between-subject mean square, *MS*_*w*_ represents the within-subject mean square at the voxel level, and *K* represents the number of sessions. Voxel-wise ICC was calculated using MATLAB 2020b (MathWorks Inc., Sherborn, MA) based on a one-way random effect with α value of 0.05 (model), single rater/measurement (type), and absolute agreement (definition). These parameters were chosen based on the terminology of McGraw and Wong (Koo and Li, 2016). For all the rs-fMRI metrics, test–retest reliability was calculated by extracting the average of each metric from the gray matter mask in both states of awake and under anesthesia. It was classified as poor (ICC < 0.4), fair (ICC = 0.4–0.55), good (ICC = 0.55–0.75), and excellent (ICC = 0.75–1.0) (Li et al., 2012; Morales et al., 2020). For the rs-fMRI metrics, voxel-wise ICC, *MS*_*b*_, and *MS*_*w*_ maps were generated. Then, we extracted the average and standard deviation (SD) of these measurements for all the rs-fMRI metrics within the gray mask for both states of awake and under anesthesia.

### Effect of scan length

To investigate the effect of scan length on the rs-fMRI metrics, for each participant, rs-fMRI data were truncated into 15 bins with *i*^*th*^ bin containing the first *i* minutes of data acquisition. Thus, 15 bins of data with a scan length ranging from 1 to 15 min were generated for each candidate for each state of awake and under anesthesia. Next, the same preprocessing steps as those applied on the entire scan mentioned above were conducted for each scan length. Furthermore, voxel-wise rs-fMRI metrics, including ALFF, fALFF, ReHo, FC, and related ICC maps were generated for each scan length.

The standardized non-linear logarithmic function curves [y = a + b ln(x)] were fitted to each ICC map generated for the rs-fMRI metrics for 15 distinct time points of the scan lengths of 1 to 15 min. Two adjustable constants including “a” and “b” variables were measured to find the optimum scan length for each metric and in two states of awake and under anesthesia with the “x” variable assigned to the time of the scan. The standardized scan length was defined as the time point where the measured derivative of the logarithmic function was at least 0.01 [(|dy/dx|) < 0.01] (Liao et al., 2013; Tomasi et al., 2017).

## Results

### Test–retest reliability of rs-fMRI metrics in awake and under anesthesia states

**Figure 2:**
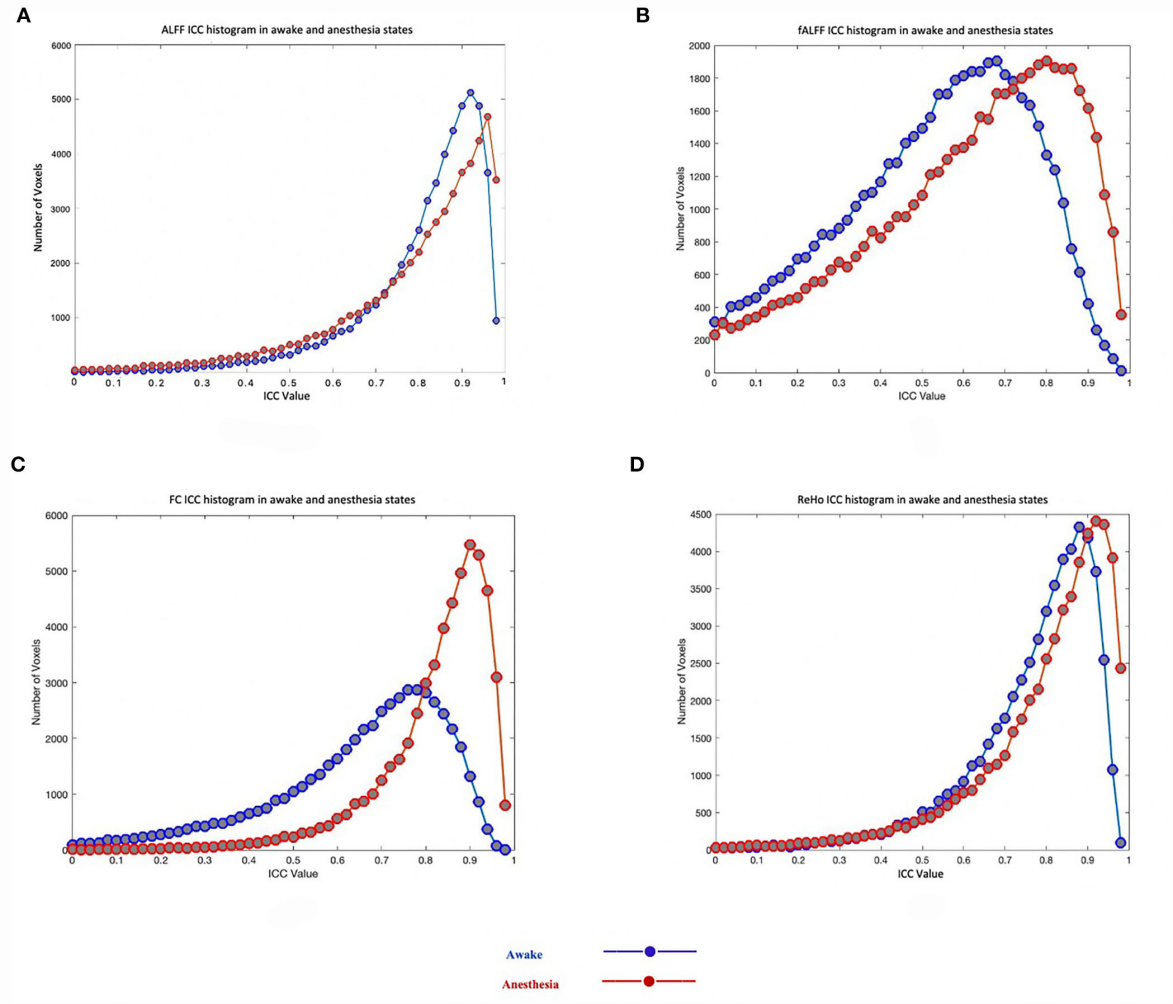
Test–retest reliability (ICC) histograms of rs-fMRI metrics including the amplitude of low-frequency fluctuation (ALFF) **(A)**, fractional amplitude of low-frequency fluctuation (fALFF) **(B)**, functional connectivity (FC) **(C)**, and regional homogeneity (ReHo) **(D)** in two states of awake and under anesthesia.

**Figure 3:**
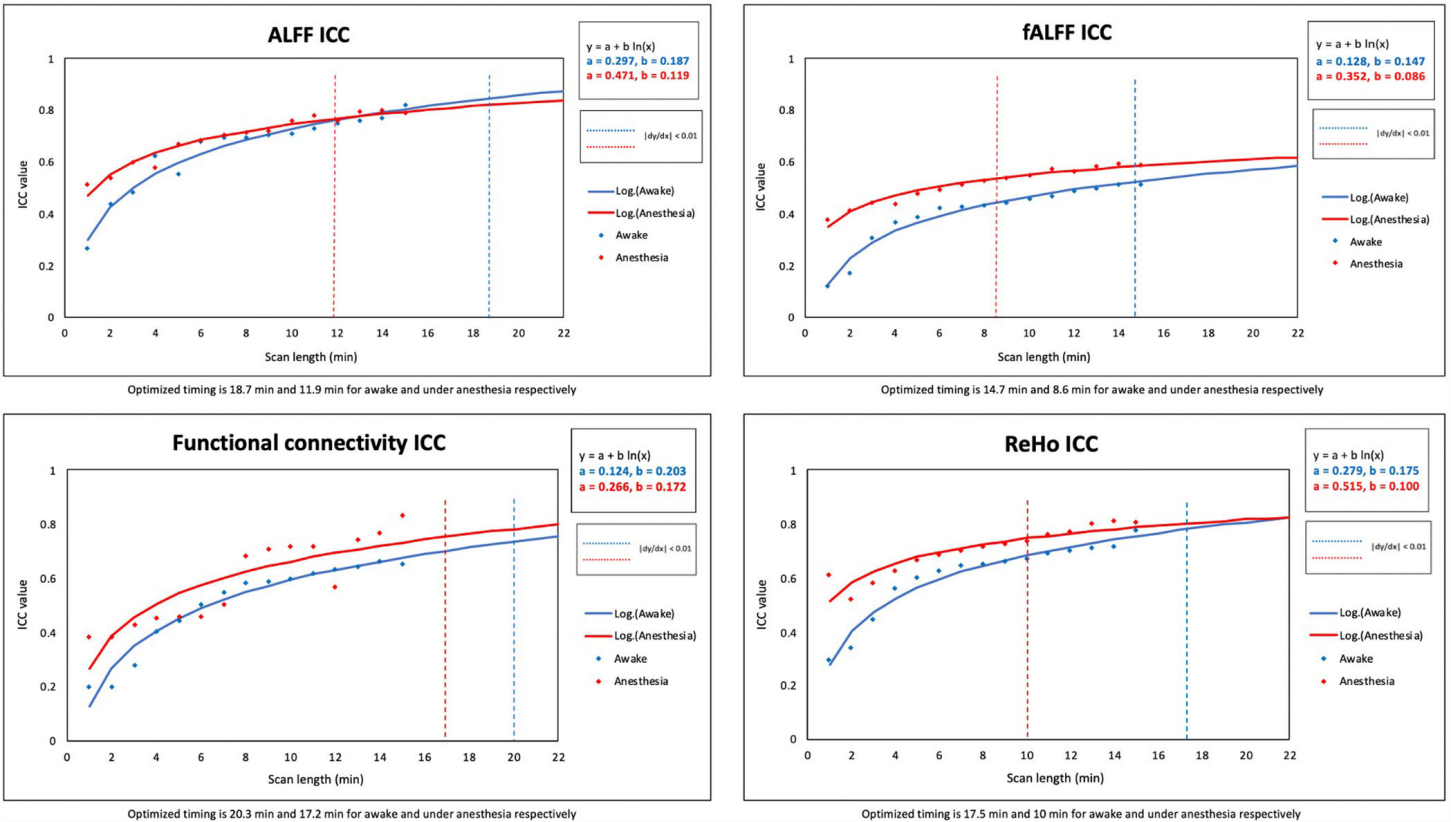
ICC maps of the amplitude of low-frequency fluctuation (ALFF), fractional amplitude of low-frequency fluctuation (fALFF), functional connectivity (FC), and regional homogeneity (ReHo) in two states of awake and under anesthesia and the difference maps between awake and under anesthesia states (blue corresponds to higher ICC in awake and red corresponds to higher ICC under anesthesia).

As shown in Table 1, the reliability of rs-fMRI metrics improved under anesthesia compared to the awake state which was much greater for FC measurement (0.65 vs. 0.83 in awake and under anesthesia, respectively). Additionally, Figure 2 shows the histograms of the ICC measurements of the rs-fMRI metrics. Brain maps of the ICC measurements are generated by BrainNet Viewer tools and are shown in Figure 3. As shown in Table 1, test–retest reliability increased for the typical ICC range (ICC <1) and excellent ICC (0.75 < ICC <1) values for fALFF and ReHo metrics in the anesthetized state compared to the awake state. Furthermore, a higher number of voxels with excellent ICC was measured for fALFF and ReHo metrics in the anesthetized state compared to the awake state (94.04 and 24.12% higher number of voxels for fALFF and ReHo, respectively). However, the number of voxels with excellent ICC was lower in the anesthetized state compared to the awake state for ALFF and FC metrics (5.17 and 18.20% lower number of voxels for ALFF and FC, respectively). This finding demonstrated that greater ICC values for FC measurement were distributed in smaller brain areas under anesthesia compared to the wakefulness state. The mean and standard deviation (SD) of ICC measurements and the number of voxels were extracted within the gray matter area of the brain (Table 1).

**Table 1:** Mean ± SD and number of voxels of ICC of rs-fMRI metrics including the amplitude of low-frequency fluctuation (ALFF), fractional amplitude of low-frequency fluctuation (fALFF), functional connectivity (FC), and regional homogeneity (ReHo) in two different masks where ICC <1 and 0.75 < ICC <1 in two states of awake and under anesthesia.

		**Number of voxels**		**MEAN ± SD**	
**rs-fMRI metric**	**ICC range**	**Awake**	**Anesthesia**	**Awake**	**Anesthesia**
ALFF	ICC < 1	54,833	54,833	0.81 ± 0.15	0.80 ± 0.20
	0.75 <ICC < 1	35,397	33,566	0.86 ± 0.06	0.88 ± 0.06
fALFF	ICC < 1	54,833	54,833	0.51 ± 0.51	0.59 ± 0.28
	0.75 <ICC < 1	9,891	19,193	0.82 ± 0.05	0.85 ± 0.06
FC	ICC < 1	54,833	54,833	0.65 ± 0.20	0.83 ± 0.13
	0.75 <ICC < 1	21,637	17,699	0.83 ± 0.05	0.84 ± 0.06
ReHo	ICC < 1	54,833	54,833	0.77 ± 0.15	0.80 ± 0.17
	0.75 <ICC < 1	28,860	35,790	0.84 ± 0.05	0.88 ± 0.06

Additionally, voxel-wise between-subject mean square and within-subject mean square maps represented by MS_b_ and MS_w_, respectively, have been generated for each rs-fMRI measurement. The mean and SD of these measurements are shown in Table 2. Under anesthesia, for all the rs-fMRI metrics, the average of between-subject variability (MS_b_) increased, and the average of within-subject variability (MS_w_) decreased compared to the values measured in the awake state which resulted in greater mean ICC values under anesthesia compared to the awake state.

**Table 2:** Mean ± SD of MS_b_ (between-subject mean square) and MS_w_ (within-subject mean square) of rs-fMRI metrics including the amplitude of low-frequency fluctuation (ALFF), fractional amplitude of low-frequency fluctuation (fALFF), functional connectivity (FC), and regional homogeneity (ReHo) in two states of awake and under anesthesia.

	**Awake**		**Anesthesia**	
**rs-fMRI metric**	**MS_b_**	**MS_w_**	**MS_b_**	**MS_w_**
ALFF	1.16 ± 1.52	0.13 ± 0.22	1.27 ± 3.60	0.08 ± 0.20
fALFF	1.07 ± 1.21	0.30 ± 0.27	1.58 ± 2.99	0.27 ± 0.33
FC	0.12 ± 0.06	0.02 ± 0.01	0.14 ± 0.02	0.01 ± 0.00
ReHo	0.52 ± 0.45	0.07 ± 0.05	0.62 ± 0.73	0.05 ± 0.04

### Optimization of scan length of rs-fMRI in awake and under anesthesia states

**Figure 4:**
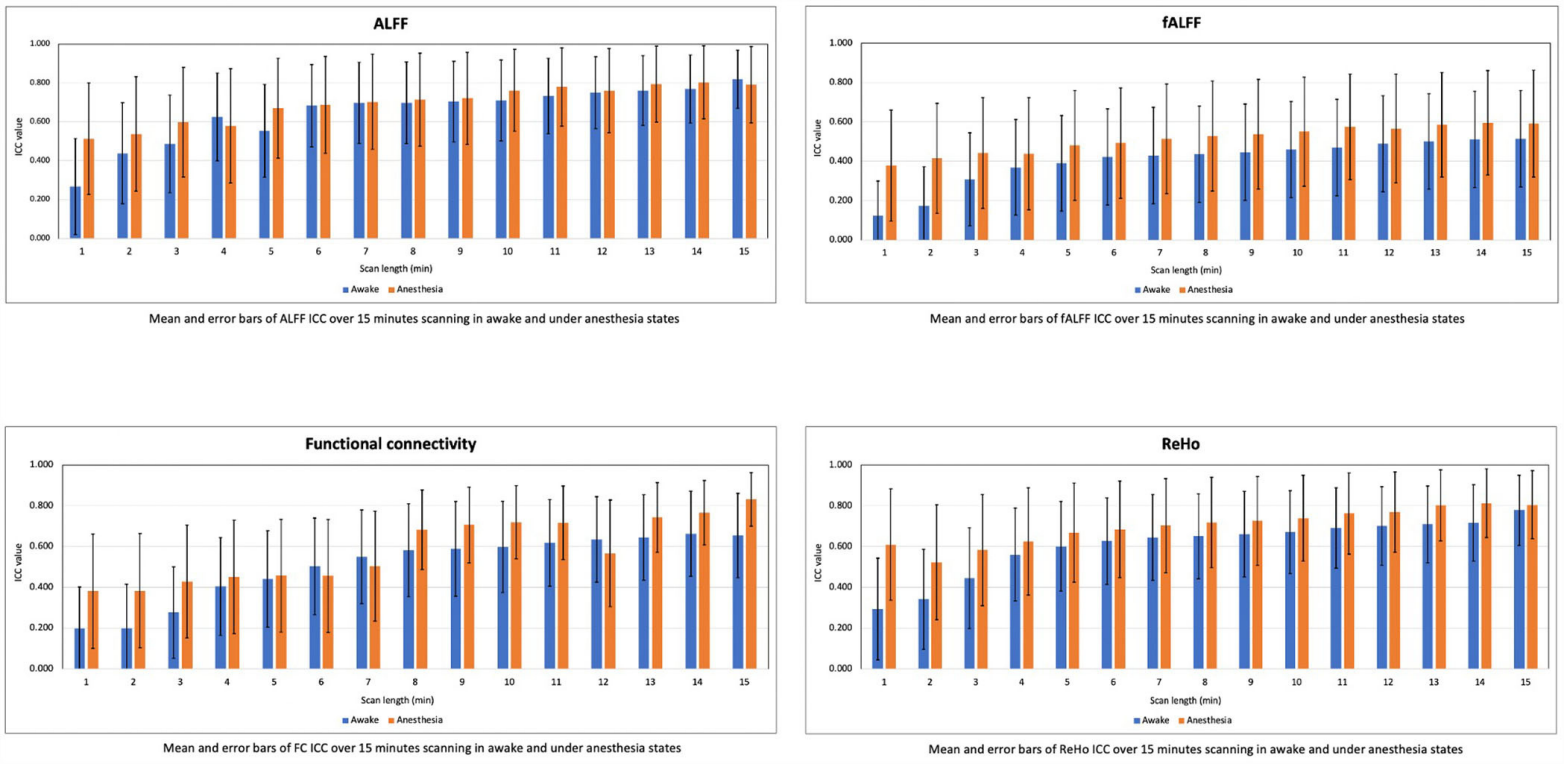
Scatter plots and standardized logarithmic fits of ICC of rs-fMRI metrics including the amplitude of low-frequency fluctuation (ALFF), fractional amplitude of low-frequency fluctuation (fALFF), functional connectivity (FC), and regional homogeneity (ReHo).

Two constant variables of “a” and “b” were calculated to find the best fit of non-linear logarithmic function for the ICC of rs-fMRI metrics in both states of awake and under anesthesia [ALFF (awake): a = 0.297, b = 0.187; ALFF (anesthesia): a = 0.471, b = 0.119; fALFF (awake): a = 0.128, b = 0.147, fALFF (anesthesia): a = 0.352, b = 0.086; FC (awake): a = 0.124, b = 0.203; FC (anesthesia): a = 0.266, b = 0.172; ReHo (awake): a = 0.279, b = 0.175; ReHo (anesthesia): a = 0.515, b = 0.100]. The “x” variable was defined as the optimum scan length where the logarithmic function reached a plateau [(|dy/dx|) <0.01]. The optimized scan lengths estimated in the awake state were 18.7 min for ALFF, 14.7 min for fALFF, 20.3 min for FC, and 17.5 min for ReHo. We found shorter optimized scan lengths in the anesthetized state for all the rs-fMRI metrics including 11.9 min for ALFF, 8.6 min for fALFF, 17.2 min for FC, and 10 min for ReHo (Table 3). Among the quantitative maps, we found the shortest optimized scan length for fALFF in both states of awake and under anesthesia. Figure 4 shows the scatter plots and logarithmic functions fitting to the ICC maps of rs-fMRI metrics and related optimized scan lengths.

**Table 3:** List of optimized scan lengths for rs-fMRI metrics at derivative logarithmic function |dy/dx| = 0.01 [y = a + b ln(x)].

**rs-fMRI metric**	**Optimized scan length in awake (min)**	**Optimized scan length under anesthesia (min)**
ALFF	18.7	11.9
fALFF	14.7	8.6
FC	20.3	17.2
ReHo	17.5	10

*ALFF, amplitude of low-frequency fluctuation; fALFF, fractional amplitude of low-frequency fluctuation; FC, functional connectivity; ReHo, regional homogeneity*.

**Figure 5:**
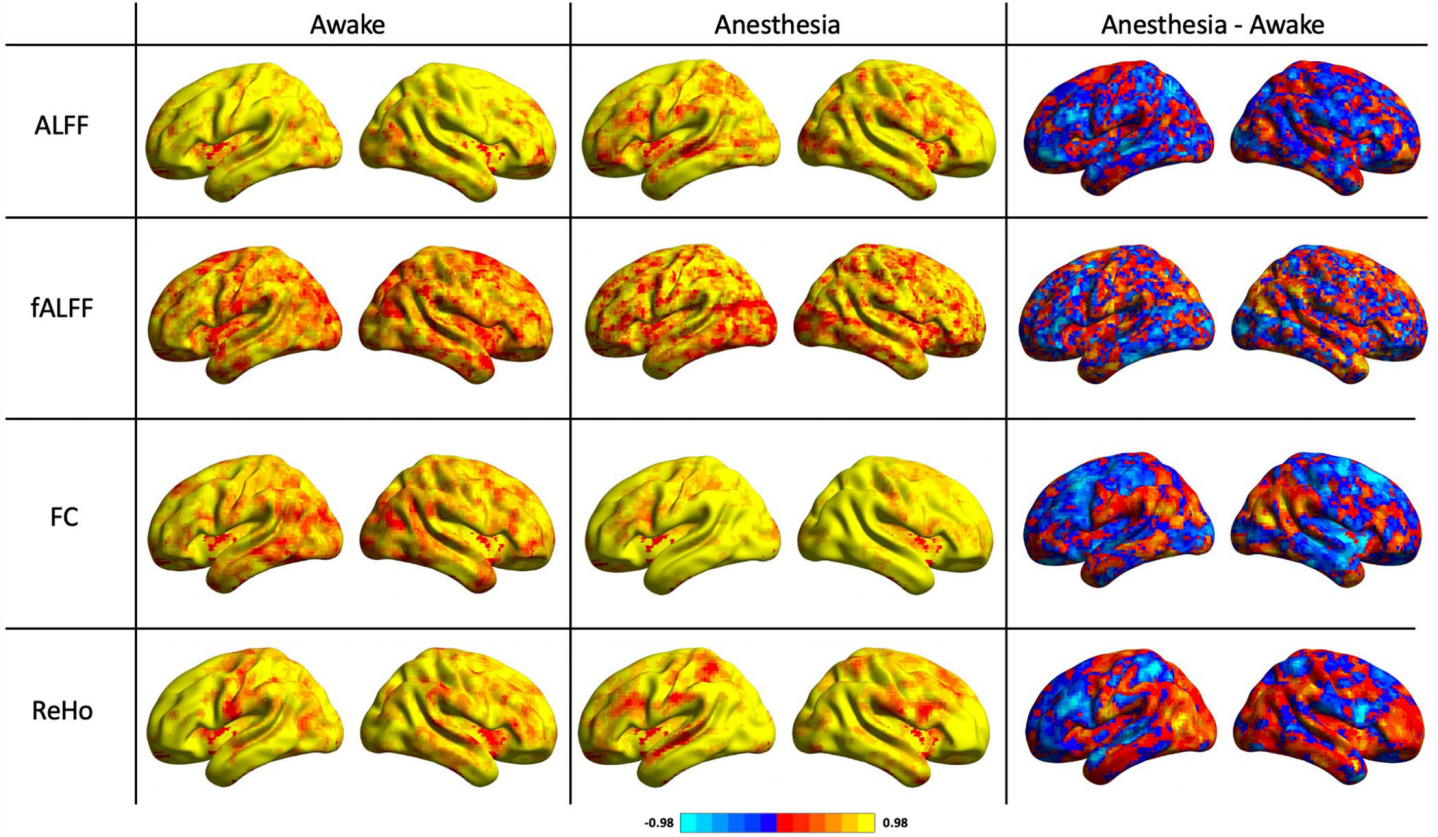
Means and error bars of ICC for the amplitude of low-frequency fluctuation (ALFF), fractional amplitude of low-frequency fluctuation (fALFF), functional connectivity (FC), and regional homogeneity (ReHo) over 1 to 15 min scan lengths.

We computed the mean and SD of ICC values for the rs-fMRI metrics in the scan lengths of 1 to 15 min within the gray matter area. As shown in Figure 5, we found that the test–retest reliability of rs-fMRI metrics increased, and variability of ICCs decreased as the function of scan length in both states of awake and under anesthesia. However, reliability was higher in the anesthetized state compared to the awake state at each time point.

## Discussion

The application of general anesthesia during rs-fMRI studies unavoidably interferes with the resting-state BOLD signal. Hereupon, understanding the confounding effects of anesthetics is of the essence in MRI acquisition, design, and data analysis in the experiments needed to get involved with anesthetics. This study provides a comprehensive evaluation of the voxel-wise test–retest reliability of rs-fMRI metrics and its relationship with scan length in two states of awake and under general anesthesia. Voxel-wise ALFF, fALFF, ReHo, and FC metrics were chosen to estimate the reliability of resting-state BOLD response as they are robust measurements and stable to noise interference. Reliable test–retest reliability of single subject metric is crucial to obtaining imaging biomarkers that assist in the detection and evaluation of developmental changes in neurological diseases. Nevertheless, test–retest reliability has been shown to be regionally variable across the whole brain. Indeed, higher-order cortical networks including default mode network, sensory/motor network, and visual network, as well as dorsal attention, ventral attention, and frontoparietal control are known to have the highest reproducibility (Somandepalli et al., 2015). Hence, the current study was limited to the gray matter area of the brain throughout the entire data processing.

Two main observations were noted. First, our findings showed an improvement in the reliability of rs-fMRI metrics measurements under anesthesia compared to the awake state that was more prominent for the scans < 12 min. Second, by modeling the non-linear logarithmic regression to the ICC maps of the rs-fMRI metrics, we showed that optimized scan length is shorter under anesthesia than in the awake state. Previous studies examined the effect of anesthesia and scan length as separate experiments on the variability of resting-state BOLD signal (Birn et al., 2013; Liao et al., 2013; Grandjean et al., 2014; Elliott et al., 2019; Becq et al., 2020). However, none has addressed this question by comparing the test–retest reliability of rs-fMRI metrics obtained in awake and under anesthesia states and combining this factor with the impact of scan length.

To that end, we compared ICC maps obtained from rs-fMRI metrics including voxel-wise ALFF, fALFF, ReHo, and FC in awake and anesthetized states. In terms of the effect of anesthesia, our findings indicated that test–retest reliability enhanced under anesthesia for the rs-fMRI metrics was much greater for FC; meanwhile, ALFF exhibited the least ICC difference between awake and under anesthesia states. Additionally, among good-to-excellent test–retest reliability of the rs-fMRI metrics, fALFF showed moderate values in both awake and anesthetized states which might be linked to the high sensitivity of this metric to detecting physiological signals compared to ALFF (Pawela et al., 2008; Zou et al., 2008; Somandepalli et al., 2015; Fang et al., 2021). These findings are consistent with the prior literature reporting that ALFF has higher test–retest reliability than fALFF. As such, it is recommended to use fALFF, or at least combine it with ALFF, in rs-fMRI studies because of the high sensitivity of this measurement to reduce physiological noises (Zuo and Xing, 2014).

### Effect of anesthesia on the test–retest reliability

Our results showed that the reliability of rs-fMRI measurements improved under anesthesia compared to the awake state. We speculate that neurophysiology characteristics of the anesthetic agents might contribute to this finding by reducing the BOLD signal variability that may lead to the enhancement of the test–retest reliability of rs-fMRI metrics under anesthesia compared to wakefulness state. It has been shown that both intravenous and inhaled anesthetic agents such as propofol and sevoflurane modulate γ-aminobutyric acid type A (GABA_A_) receptors, which is the fastest inhibitory neurotransmitter receptor in the central neural system. Especially, propofol one of the potent modulators of GABA_A_ receptors enhances the gating of the receptors and thereby reduces neural excitability. The volatile anesthetics including sevoflurane enhances GABA_A_ receptor function which leads to increasing channel opening and inhibition enhancement at both synaptic and extrasynaptic receptors by reducing neural excitability (Hemmings et al., 2005; Franks, 2006). Such reduction in the receptive field size under anesthesia has been revealed for somatosensory cortical and sub-cortical neurons which suppress consciousness through actions that control sleep-wake states (Mashour, 2014; Aksenov et al., 2015; Moody et al., 2021). However, anesthetics may also change neurovascular physiology and cerebrovascular reactivity which in turn can alter the BOLD signal without any underlying changes in the brain activity and ultimately change the test–retest reliability of measured fMRI signal (Masamoto and Kanno, 2012; Aksenov et al., 2015).

Our results were qualitatively comparable to the results from previous literature finding that anesthesia reduces temporal variability across cortical regions during the loss of consciousness that was measured by the SD of BOLD response (Pawela et al., 2008; Huang et al., 2014, 2016; Bettinardi et al., 2015). A recent animal study comparing BOLD signal variability in awake and anesthetized rats showed the reduction of variability across much of the brain under anesthesia. They proposed that variability can be used as a robust signature of consciousness that distinguishes anesthesia-induced unconsciousness from the awake state (Baria et al., 2018).

However, this is not necessary that reduced BOLD response variability improves test–retest reliability. In fact, reduced BOLD response variability can also co-occur with reduced test–retest reliability if differences between subjects are decreased. It has been shown that test–retest reliability improves as subjects become more distinct from each other and/or as within-subject measurements become more similar (Noble et al., 2021). In line with this, our findings demonstrated that under anesthesia temporal between-subject variability increases and within-subject variability decreases which results in greater test–retest reliability of ICC measurements (Table 2).

Several factors may impact the reliability of resting-state BOLD response, and as such, test–retest reliability can vary fundamentally between datasets ranging from poor-to-excellent ICC. We note that since head motion during scanning can affect test–retest reliability that was intrinsically restricted under anesthesia, higher-order regression models including the Friston 24-parameter model were employed at the individual level to minimize the micro head motion artifacts in both states of awake and under anesthesia (Friston et al., 1996; Guo et al., 2012; Zuo and Xing, 2014; Ciric et al., 2018; Mahadevan et al., 2021). However, it was intrinsically restricted under anesthesia. In addition, other variations related to non-neuronal physiology, such as white matter (WM) and cerebrospinal fluid (CSF), that represent nuisance signals were excluded as covariate factors in both states (Parkes et al., 2018). Therefore, it is unlikely that the results of ICC differences between awake and under anesthesia reflected motion artifacts and other physiological noises since data underwent the same image processing steps in both states. Rather, in interpreting our findings, we affirm that the improvement of test–retest reliability under anesthesia might be associated with the suppression of neural factors contributing to resting-state BOLD response and cerebrovascular reactivity, as well as temporal within-subject and between-subject variabilities. Additionally, due to the good test–retest reliability of rs-fMRI metrics including ALFF, FC, and ReHo measured in the current study, our findings propose that these rs-fMRI metrics can potentially be used as robust imaging biomarkers in rs-fMRI studies in either awake or under anesthesia.

### Effect of scan length in awake and anesthetized states

Numerous studies have proven that scan length is one of the key parameters in the design of MRI scanning that plays an important role in generating robust and stable results, particularly in experiments where candidates are anesthetized or have difficulty staying during scanning. They have reported the appropriate scan duration to achieve a stable brain's functional connectivity in the wakeful state (Wang et al., 2011, 2021; Birn et al., 2013; Liao et al., 2013; Mejia et al., 2018; Teeuw et al., 2021). For instance, Birn et al. (2013) showed that the test–retest reliability of FC can be significantly improved by increasing the duration of scanning to 9–13 min or longer (Birn et al., 2013). In line with this, a previous study using a machine-learning classifier suggested that a minimum of 15–25 min of rs-fMRI data in a single subject is required to obtain moderate reproducibility of quantitative FC (Anderson et al., 2011). Consistent with these, another study in the context of seed-based FC within resting-state networks (RSN) examined the effect of scan duration using nine distinct time points (3–27 min) on the test–retest reliability of fMRI signal and found improvement of ICC over time, until the time point where the plateau reached around 12–16 min and 8–12 min for intrasession and intersession, respectively (Birn et al., 2013). Additionally, a recent study on Human Connectome Project (HCP) computed ICC for rs-fMRI graph metrics. Depending on the sample size and the number of time points (duration of scan), they found that for a large sample size (for instance, 100 subjects), most of the global and regional graph metrics were reliable for a minimum scan duration of 7 min. Additionally, for a small sample size (for instance, 40 subjects), they found that most of the global graph metrics were reliable in a longer scan duration of at least 14 min. However, at the regional level graph, metrics were reliable in the areas located in the default mode network, visual, and motor areas (Termenon et al., 2016).

While previous studies estimated the optimized scan duration to obtain reliable resting-state function connectivity measures in wakefulness state, our study is the first standardizing scan length of the rs-fMRI studies in both anesthetized and wakefulness states using the test–retest reliability of several rs-fMRI metrics. In this study, rs-fMRI metrics were computed in 15 distinct time points (with 1 min interval between) of 15-min scan length individually in both states of awake and under anesthesia. For each metric, ICC maps of rs-fMRI metrics over 15-time points were fitted to the logarithmic function using non-linear regression [y = a + b ln (x), where x is defined as scan duration (min)]. The optimized scan duration was determined at the time where the standardized function reached the plateau [(|dy/dx|) < 0.01]. Our results agree well with previous literature finding that test–retest reliability improves as scan duration increases (Wang et al., 2011). Furthermore, we propose that resting-state scans under anesthesia require a shorter scan length to achieve a less variable response compared to scans in the awake state [(|dy/dx|) < 0.01]. According to the optimized scan length calculated for distinct rs-fMRI metrics, we suggest that optimized scan length within the range of 14.7–20.3 min and 8.6–17.2 min for rs-fMRI scans in awake and under anesthesia, respectively, is required to ensure less variability of rs-fMRI measurements [(|dy/dx|) < 0.01]. Our findings agree well with the previous study, suggesting that the reliability of FC can be improved by increasing the imaging duration to 13 min and longer in the awake state (Birn et al., 2013). Moreover, our results provided evidence that general anesthesia has a significant effect on the reliability of the resting-state metrics resulting in shorter optimized scan length compared to the awake state. Additionally, we showed that among the quantitative maps, fALFF reached the optimized ICC in shorter scan duration (8.6 min under anesthesia and 14.7 min in awake) and FC in longer scans (17.2 min under anesthesia and 20.3 min in awake). Our study translates these findings into a real-world clinical setting in which it may assist in the determination of the appropriate imaging biomarker in rs-fMRI studies with different scan lengths.

This study is the first to introduce a systematic approach to optimize rs-fMRI scan length for either wakefulness or an anesthetized state that can be helpful to prognosticate the trend of ICC measurements over time according to the modeled logarithmic functions. We employed brain LFO measurements at rest including ALFF, fALFF, ReHo, and FC in both states of awake and under anesthesia and defined the optimized scan length that can assure reliable responses. However, prior analytical studies employed different strategies to determine the optimized scan duration and just in the awake state (Birn et al., 2013; Andellini et al., 2015; Choe et al., 2017; Zhang et al., 2018).

There are some potential limitations involved in our study. First, this is important to keep in mind that resting-state BOLD response may be modulated by the number of participants included in the study. Previous studies showed that poor reproducibility is associated with a small sample size due to the low statistical power of ICC measurements. In fact, sample size defines the number of degrees of freedom, which is a key element in determining the statistical power of ICC measurement at a group level. They reported the minimum sample size of 20 subjects that allows reliable rs-fMRI measurements (Thirion et al., 2007; Button et al., 2013; Termenon et al., 2016). As such, a trade-off between the number of subjects and scan length is necessary to achieve the high reproducibility of outcomes. Hence, our findings suggest that the smaller sample size (below 40 subjects) requires a longer scan duration to obtain reproducible results as we reported in our experiment. Future research is needed to take this into account by investigating the reliability of fMRI signals with larger sample size and different time points. Taking together, the number of factors including sample size, scan duration, and effect of general anesthesia play key roles in the reliability of rs-fMRI measurements that can be optimized through an appropriate trade-off between these parameters.

Second, this study measured ICC values of rs-fMRI metrics within the gray matter area. Further studies are needed to investigate the test–retest reliability of rs-fMRI metrics in both states of wakefulness and under anesthesia within distinct brain networks. Additionally, we note that this study was limited to the total scan duration of 15 min. By modeling the non-linear logarithmic regression to the ICC maps, we showed that for all the rs-fMRI metrics at different time points, ICC is higher under anesthesia than in the awake state. However, the trend of ICC values may change beyond 15 min. Indeed, ICC may drop under anesthesia after a while at specific time. Whereas, at the same time ICC is still consistent in awake state. This conclusion is convincing and commensurate with the pathophysiology of anesthetics in which over time the effect of the anesthetics may be diminished, or physiological noise may generate fluctuations and intervene in the estimated fMRI response. Furthermore, it is important to note that this study was focused on the scans acquired in a distinct repetition time (TR) or the number of volumes per specific time. However, scan duration and the number of acquired volumes are mutually dependent. Moreover, different TRs may impact the BOLD signal due to different factors involved, such as the time of recovery and in-flow effect. Thus, shorter TR (rapid imaging pulse sequences) would improve not only the efficiency of detecting connections but also enhance the test–retest reliability of estimated rs-fMRI BOLD signal that can be investigated by future research.

Lastly, we need to point out that patients with epilepsy comprise a heterogeneous population with regard to pathophysiology, location, and extent of the epileptogenic zone and with respect to the various anti-epileptic medications used to treat seizures. The different clinical presentations may represent different patterns of seizure spread or different extent of involvement of the network which could affect the reliability of the resting state when combined. Therefore, the identification of differences in neuroplasticity might be biased by these clinical presentations. This limitation can be addressed in future studies by including a sample large enough to cluster patients according to the specific type of seizure. Although we are confident that the proposed study worked reasonably well in the current patient group, where patients only had unilateral focal epilepsy.

While previous studies have shown rs-fMRI as a biomarker of brain function that can be employed in medical diagnosis, our results in line with prior literature are promising in the design of longitudinal studies with disease progression. Gains in intersession/intrasession reliability of rs-fMRI measurements could be important for the interpretation of longitudinal fMRI studies. Other measures, such as improved session-to-session alignment and noise correction techniques, are necessary to improve intersession reliability, which is critical for longitudinal studies.

With increasing interest in reproducible findings using rs-fMRI, there has been a growing number of studies evaluating the reliability of rs-fMRI typically measured by the ICC. Some recent studies reported the number of factors that play the main roles to boost the reproducibility of the measurements including study design and analysis decisions. Our results contribute to the body of research standardizing the reliability of rs-fMRI metrics through combining factors including the application of anesthetic agents and scan duration. In the present study, we comprehensively examined the reliability of rs-fMRI metrics within spontaneous low-frequency fluctuations (0.01–0.08 Hz) of brain activity from scans up to 15 min in duration. Our findings demonstrated the improvement in the reliability of rs-fMRI responses under anesthesia compared to the awake state across distinct time points. Additionally, we revealed that a shorter acquisition time is required to achieve comparable reproducibility under anesthesia. This systematic exploration of the reliability of rs-fMRI measures is helpful in the implication of anesthetic agents in clinical practice and longitudinal studies of patients who need to get scanned under anesthesia.

## Data availability statement

The raw data supporting the conclusions of this article will be made available by the authors, without undue reservation.

## Ethics statement

The studies involving human participants were reviewed and approved by Thomas Jefferson University Hospital. The patients/participants provided their written informed consent to participate in this study. Written informed consent was obtained from the individual(s) for the publication of any potentially identifiable images or data included in this article.

## Author contributions

FV: conceptualization, methodology, validation, formal analysis, investigation, resources, data curation, writing—review and editing, visualization, supervision, and project administration. MA: methodology, validation, formal analysis, investigation, data curation, and writing—review and editing. FM: conceptualization, methodology, data curation, writing—review and editing, supervision, and project administration. CW: conceptualization, methodology, validation, resources, project administration, funding acquisition, investigation, resources, and writing—review and editing. All authors reviewed the manuscript. All authors contributed to the article and approved the submitted version.

## Conflict of interest

The authors declare that the research was conducted in the absence of any commercial or financial relationships that could be construed as a potential conflict of interest.

## Publisher's note

All claims expressed in this article are solely those of the authors and do not necessarily represent those of their affiliated organizations, or those of the publisher, the editors and the reviewers. Any product that may be evaluated in this article, or claim that may be made by its manufacturer, is not guaranteed or endorsed by the publisher.
