# Supplementary material for: The effect of general anesthesia on the test–retest reliability of resting-state fMRI metrics and optimization of scan length
Source: Front Neurosci. 2022 Aug 16;16:937172. doi: 10.3389/fnins.2022.937172 (PMC9425911; doi:10.3389/fnins.2022.937172)
Supplement: Supplementary file 1 [file Table_1.DOCX]

***Motion parameters in awake and under anesthesia states:***

Higher average and standard deviation (SD) of mean framewise displacement (FD) were measured in awake compared to under anesthesia state (awake: 0.155 ± 0.120, anesthesia: 0.101 ± 0.028 (mean ± SD)).

Mean framewise displacement (FD)-Jenkinson for each patient in both sessions of scanning and two states of awake and under anesthesia.

| subjects | Awake 1 | Awake 2 | Anesthesia 1 | Anesthesia 2 |
| --- | --- | --- | --- | --- |
| 1 | 8.82E-02 | 1.16E-01 | 1.26E-01 | 1.25E-01 |
| 2 | 1.66E-01 | 1.08E-01 | 1.18E-01 | 1.06E-01 |
| 3 | 1.88E-01 | 4.03E-01 | 8.83E-02 | 8.15E-02 |
| 4 | 4.87E-01 | 1.50E-01 | 1.26E-01 | 1.20E-01 |
| 5 | 3.86E-02 | 3.90E-02 | 6.28E-02 | 6.39E-02 |
| 6 | 2.02E-01 | 2.30E-01 | 1.08E-01 | 1.38E-01 |
| 7 | 9.56E-02 | 1.30E-01 | 8.21E-02 | 8.49E-02 |
| 8 | 9.83E-02 | 1.49E-01 | 6.00E-02 | 6.25E-02 |
| 9 | 4.90E-02 | 5.25E-02 | 1.27E-01 | 1.43E-01 |
